# Oleic Acid Status Positively Correlates with the Soluble Receptor for Advanced Glycation End-Products (sRAGE) in Healthy Adults Who Are Homozygous for G Allele of RAGE G82S Polymorphism

**DOI:** 10.3390/cells12121662

**Published:** 2023-06-19

**Authors:** Permal Deo, Varinderpal S. Dhillon, Philip Thomas, Michael Fenech

**Affiliations:** 1 Health and Biomedical Innovation, UniSA Clinical and Health Sciences, University of South Australia, Adelaide 5000, Australia; 2 CSIRO Health and Biosecurity, Adelaide 5000, Australia; 3 Genome Health Foundation, North Brighton 5048, Australia

**Keywords:** advanced glycation end products (AGEs), soluble receptor for AGEs (sRAGE), red blood cell (RBC) fatty acids, monounsaturated fatty acid (MUFA)

## Abstract

Background: The soluble form of receptor for advanced glycation end products (sRAGE) have been implicated in the prevention of numerous pathologic states, and highlights as an attractive therapeutic target. Because diets rich in monounsaturated fatty acids (MUFA) reduce postprandial oxidative stress and inflammation that is related to better health during aging, we investigated the association between red blood cell (RBC) fatty acids with circulatory AGE biomarkers and further stratified this correlation based on GG and GA + AA genotype. Methods: A total of 172 healthy participants (median age = 53.74 ± 0.61 years) were recruited for the study. RBC fatty acid was analysed using gas chromatography and sRAGE was measured using a commercial ELISA kit. Results: The result showed a non-significant correlation between total MUFA with sRAGE however oleic acid (C18:1) exhibited a positive correlation (r = 0.178, *p* = 0.01) that remained statistically significant (β = 0.178, *p* = 0.02) after a stepwise multivariate regression analysis after adjusting for age, BMI and gender. In a univariate analysis, a positive significant correlation between C18:1 and sRAGE in GG genotype (r = 0.169, *p* = 0.02) and a non-significant correlation with GA + AA genotype (r = 0.192, *p* = 0.21) was evident. When C18:1 was stratified, a significant difference was observed for oleic acid and G82S polymorphism: low C18:1/GA + AA versus high C18:1/GG (*p* = 0.015) and high C18:1/GA + AA versus high C18:1/GG (*p* = 0.02). Conclusion: Our study suggests that increased levels of C18:1 may be a potential therapeutic approach in increasing sRAGE in those with GG genotype and play a role in modulating AGE metabolism.

## 1. Introduction

Advanced glycation end products (AGEs) and receptors for advanced glycation end-products (RAGE) have been implicated in a range of inflammatory diseases including cardiovascular, diabetes, Alzheimer’s disease, and cancers. AGEs, a group of post-translational modifications, are produced via non-enzymatic reaction by oxidation, rearrangement, and cross-linking between the active carbonyl groups of reducing sugars and the free amines of the amino acids [1,2]. Most AGEs exhibit fluorescent, crosslinked (e.g., pentosidine and crosslines), or non-fluorescent, non-crosslinked, [e.g., N*^ε^*-carboxymethyllysine (CML), N*^ε^*-carboxyethyllysine (CEL), and N*^δ^*-methylglyoxal-derived hydroimidazolones (MG-H1)], or non-fluorescent, crosslinked structures (e.g., di-carbonyl-lysine dimers) [3].

AGEs impact tissue damage via a receptor-dependent mechanism which comprises alteration of cellular functions by binding with the full-length membrane-bound RAGE. RAGE, a multiligand signal transduction receptor, could bind with ligands such as AGEs, S100, and β-amyloid (Aβ) [4,5]. The AGE/RAGE interactions induce activation of nuclear factor-kappa beta (NF-κB) synchronised inflammatory pathway which increases inflammatory cytokine production and reactive oxygen species thus associated with the progression of disease [5,6]. In contrast, the soluble form of RAGE, communally named sRAGE, are produced either by proteolytic cleavage (cRAGE) or by alternative splicing (esRAGE) and plays an antagonistic role by competing for AGEs with the cell surface full-length RAGE, consequently preventing the induction of RAGE arbitrated inflammatory response within cells [4,6].

The genetic variability of RAGE results from single-nucleotide polymorphism (SNPs) and over 30 SNPs identified within the RAGE gene are associated with the development of human diseases such as diabetic complications, cancer, and Alzheimer’s disease [7]. Of these SNPs, the RAGE genetic variant rs2070600 (Gly82Ser, G82S), which causes a glycine (G) to serine (A) substitution at amino acid number 82 of the ligand-binding V-domain in the extracellular domain, has been associated with circulating AGEs and sRAGE levels [8,9,10]. Consequently, this RAGE variant displays enhanced ligand binding to AGEs that are associated with increased NF-κB activation and inflammatory gene expression [8,10]. In addition, the G82S polymorphism is associated with a reduced level of sRAGE however, how ligand binding and sRAGE levels are altered in this RAGE variant is unknown [7].

Proper diet is a key component of a healthy lifestyle and in the prevention and management of diseases. The beneficial effect of monounsaturated and polyunsaturated fatty acids are widely reported [11,12]. Mediterranean diet, rich in monounsaturated fatty acids (MUFA) and includes minimally processed natural foods, reduces postprandial oxidative stress and inflammation and is thus associated with healthy aging [13,14]. Further, consumption of MUFA-rich diets are associated with a reduction in serum AGEs, RAGE mRNA expression, oxidative stress biomarkers, and increased AGE Receptor 1 (AGER1) and glyoxalase 1 enzyme (Glox 1) gene expression [15,16]. Oleic acid, a MUFA, a major fatty acid in dietary fat and plasma triglycerides is a key Mediterranean diet component that has been associated with the prevention and management of many chronic diseases including atherosclerosis and diabetes [17,18,19].

In a previous study, we explored the association of saturated and unsaturated sub-classes of red blood cell (RBC) fatty acids and AGE biomarkers [20]. In the present study, we tested the hypothesis that the MUFA subclass of RBC fatty acids was correlated with sRAGE, and this association was influenced by RAGE G82S polymorphism.

## 2. Materials and Methods

### 2.1. Study Participant Recruitment

Volunteers were recruited through the following method: (i) hospitals and universities posted advertisements within the Adelaide metro area (ii) the CSIRO Clinical Research Unit database in Adelaide, and (iii) Channel 7 news report of this study. One hundred and seventy-two healthy participants (35–65 years old) were recruited who passed the inclusion criteria: non-smokers, not currently diagnosed with mild cognitive impairment (MCI) or AD, mini-mental state examination (MMSE) score ≥ 20, not on medication for life-threatening diseases (e.g., chemotherapy), not taking daily minerals, fish oil, and vitamin supplements above the RDA level, able to understand the study protocol and are not on cholesterol-lowering medication.

### 2.2. Blood Collection, Lipid, and RBC Fatty Acid Analysis

For lipids and AGE biomarker analysis, 12 h fasted whole blood samples were collected at the clinic via venipuncture in lithium-heparin (LH) (Greinder Bio-One, Melbourne, Australia) vacutainer tubes, and samples were centrifuged at 1500× *g* for 20 min at 4 ± 1 °C, plasma removed and stored at −80 °C until further analysis. Triglycerides, total cholesterol, HDL-cholesterol, and LDL-cholesterol were measured on the Hitachi 902 Automated Clinical Analyser at the CSIRO Analytical and Clinical Chemistry Laboratory in Adelaide. For RBC fatty acid analysis, 8 mL of 12 h fasted whole blood samples were collected in ethylenediamine tetra-acetic acid (EDTA) tubes processed and analysed as described elsewhere [20,21].

### 2.3. DNA Isolation and RAGE G82S Gene Polymorphism

Lymphocytes were isolated from the fresh blood and stored at −80 °C until DNA was isolated for RAGE G28S gene polymorphism assay. Genomic DNA was isolated from lymphocytes using a Qiagen DNeasy Blood and Tissue kit according to the manufacturer’s instructions (Qiagen, Hilden, Germany). Commercially available TaqMan^®^ SNP Genotyping Assay probes supplied by Applied Biosystems were used to determine RAGE G28S polymorphism. Polymorphism in the RAGE gene (G82S; rs2070600; C>T) was performed using TaqMan technology on an ABI7300 system (Applied Biosystems, Carlsbad, CA, USA) as previously described [22]. Briefly genotyping for RAGE was determined using a commercially available polymerase chain reaction (PCR) assay. The PCR reaction included TaqMan SNP Genotyping Assay reagents (C_15867521_20; Life Technologies, Waltham, MA, USA), genomic DNA (2.0 g/mL), TaqMan Universal PCR Master Mix (Life Technologies Corp) and standard polymerase chain reaction (PCR) and was performed in a 10 μL volume.

### 2.4. Glucose and Glyoxal Analysis

Plasma glucose concentration was analysed using hexokinase method with Konelab System reagents and measured by an automated spectrophotometric analyser (Konelab 20Xti, Thermo Electron, Waltham, MA, USA). Girard’s reagent T was used to determine the glyoxal concentration as described previously [23]. Briefly, aliquots of samples (100 μL), Girard-T solution (25 μL, 500 mM), and sodium formate (125 μL, 500 mM, pH 2.9) were added to a 96 well plate before 1 h incubation at room temperature in the dark. Absorbance was measured at 285 nm using a multimode microplate reader (EnSpire^®^ Perkin Elmer, Waltham, MA USA). Glyoxal (Sigma-Aldrich, Sydney, Australia) was used as standards (0–50 nmol/L) and results are expressed as nmol/L.

### 2.5. Total Fluorescent AGEs, CML Levels and sRAGE Analysis

Fluorescent AGEs were measured as reported elsewhere [20,23]. Briefly, 250 μL plasma (50-fold dilution in 10 mM phosphate buffer saline (PBS, pH 7.2)) was aliquoted into black 96-well plates and the fluorescence (370 nm Ex: 440 nm Em) was measured using a multimode microplate reader (EnSpire^®^ Perkin Elmer, Waltham, MA, USA). The fluorescence of PBS (pH 7.2) alone was subtracted to obtain measurements in arbitrary units (AU). Results are presented as the mean of triplicate reading for each sample and expressed as AU/mL.

CML content in samples was determined by RP-HPLC with an o-phthaldialdehyde derivatisation step as described previously [20,23]. Briefly, plasma (100 μL) was initially reduced with sodium borohydride before protein isolation with trichloroacetic acid (20%) and then hydrolysed with hydrochloric acid (6 M) at 110 °C for 24 h [24]. After derivatisation, samples were injected (15 μL) onto a RP C18 HPLC column (Aeris™, 2.1 × 150 mm, particle size 3.6 μm, pore size 200Å; Phenomenex, NSW, Australia) and monitored with a fluorescence detector at 340 nmEx/450 nmEm. The levels of lysine and CML were determined from a 5-point calibration standard curve of lysine (Sigma, St. Louis, MO, USA) and CML (Polypeptide Laboratories, France) respectively. The standard was measured in triplicate, while samples were measured in duplicates. Results are presented as μmol CML/mol Lysine.

Total soluble extracellular domain of RAGE (sRAGE) concentration in plasma was determined using a commercial ELISA kit (BioVendor, Karasek, Czech Republic). The assay measured total sRAGE resulting from both cleavage (cRAGE) and endogenous secreted (esRAGE) of RAGE and measured as per the manufacturer’s instructions. The intra-assay and inter-assay coefficients of variation values were <5% and <10%, respectively.

### 2.6. Statistical Analysis

Parametric or non-parametric tests were used based on the data as with normal Gaussian distribution or non-Gaussian distribution, respectively. Correlation analysis was performed by Pearson’s test. Multivariate linear regression analysis (Stepwise) with age, BMI, and gender as confounding factors were used to search for independent associations between AGE biomarkers and RBC fatty acids. All statistical analyses were performed using GraphPad Prism 8.0 (GraphPad Inc., San Diego, CA, USA) or SPSS (version 25, IBM, Armonk, NY, USA) for one-way ANOVA and multiple regression models, including general linear modelling. Statistical significance was set at *p* < 0.05.

## 3. Results

### 3.1. Study Population Characteristics

This study consisted of 172 volunteers with 36 males and 136 females, a mean age of 53.74 ± 0.61 years, and a mean BMI of 26.52 ± 0.37 kg/m^2^ (Table 1). The plasma lipid profile and glycation biomarkers in this cohort are presented in Table 1.

For RAGE G82S polymorphism, the cohort consisted of 88% dominant homozygous genotype (GG), 10.5% heterozygous genotype (AG), and only 1.2% mutant homozygous genotype (AA). For comparison analysis, AG + AA genotype (n = 20) were combined and compared with the GG genotype (n = 152) and presented in Table 2. The sRAGE levels were significantly lower in AG + AA genotype group compared to the GG genotype (517.38 ± 35.77 pg/mL versus 677.77 ± 24.57 pg/mL, *p* = 0.001; Table 2). Glucose, glyoxal, fluorescent AGEs, and CML showed a non-significant increase in the AG + AA genotype group when compared with the GG genotype group (Table 2).

RBC’s mean and interquartile range of the individual fatty acids of the study cohort are presented in Supplementary Table S1 which contained a subclass of 52% saturated fatty acids (SFA), 21% MUFA, and 27% PUFA, respectively. Oleic acid (C18:1) was the most abundant (68%) among the MUFA subclass (Supplementary Table S1).

### 3.2. Association of RBC C18:1, MUFA and Total sRAGE

**Figure 1:**
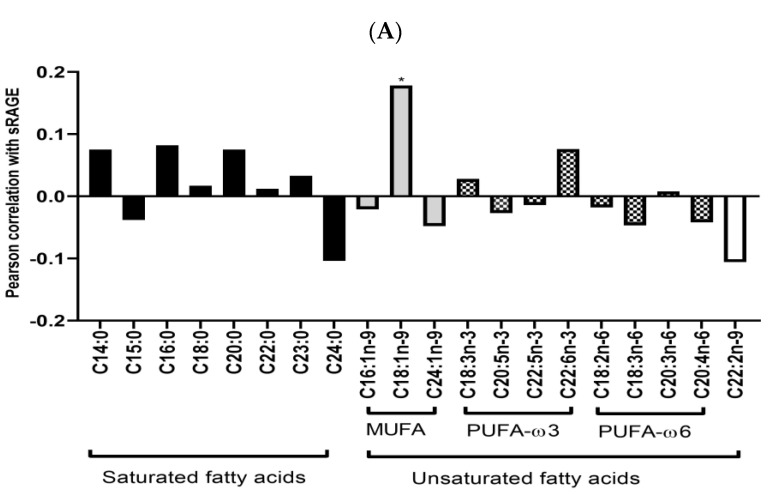

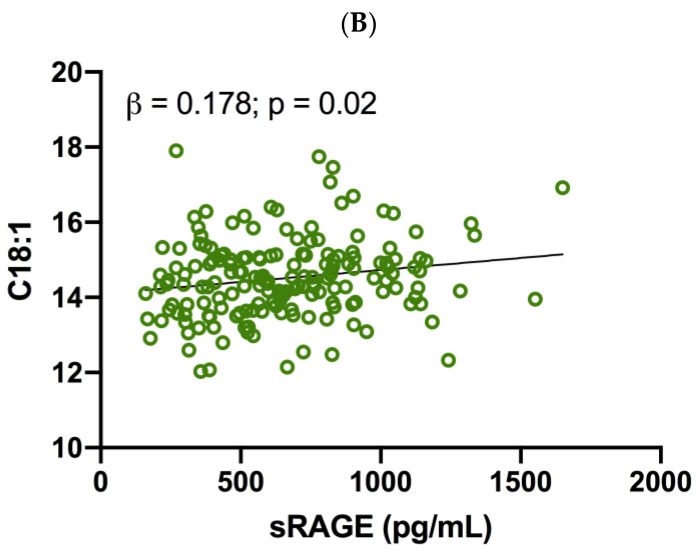
Pearson’s correlation and regression analysis for all red blood cell fatty acids with sRAGE. (**A**) Pearson’s correlation. * *p* = 0.01. Fatty acid names are given in Supplementary Table S1. MUFA = monounsaturated fatty acids; PUFA-ω3 = ω3-polyunsaturated fatty acids; PUFA-ω6 = ω6-polyunsaturated fatty acids. (**B**) Regression analysis of oleic acid (expressed as % of total RBC fatty acids) with sRAGE adjusted for age, BMI, and gender.

Univariate analysis between RBC FA and plasma sRAGE showed no statistical significance for SFA, other MUFA, or PUFA except for oleic acid (C18:1) which showed a positive correlation C18:1 (r = 0.178, *p* = 0.01; Figure 1A). A stepwise multivariate regression analysis of RBC fatty acids further confirmed a positive correlation for C18:1 with sRAGE (β = 0.178, *p* = 0.02; Figure 1B) after adjusting for age, BMI, and gender. In further analysis, a non-significant correlation for total MUFA (C16:1, C18:1, C24:1) with sRAGE (β = 0.096, *p* = 0.21) was evident (Supplementary Figure S1A).

### 3.3. Relationship between C18:1 and RAGE G82S Polymorphism

**Figure 2:**
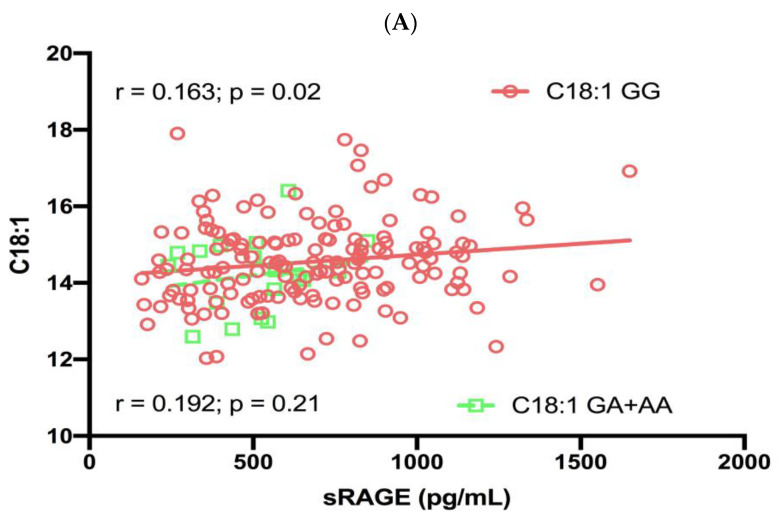

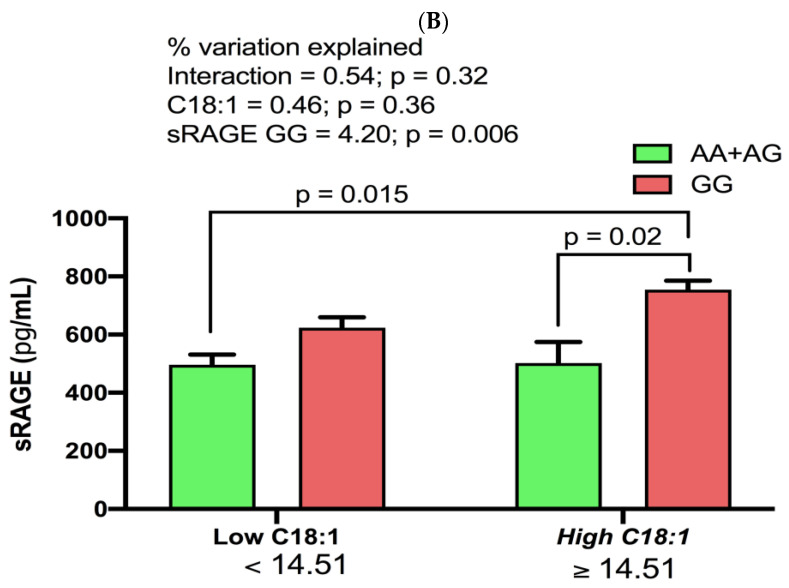
Regression analysis and ANOVA analysis. (**A**) Regression analysis of oleic acid (expressed as % of total RBC fatty acids) with RAGE genotype; (**B**) Two-way ANOVA analysis on the effect of oleic acid (high or low) on sRAGE based on RAGE genotype (AA+ AG or GG). Only significant *p*-values are indicated. Other comparisons were not significant.

Figure 2 shows the correlation and 2-way ANOVA analysis for the relationship between C18:1 and RAGE G28S polymorphism. Univariate analysis showed a positive significant correlation between C18:1 and sRAGE in the GG genotype group (r = 0.163, *p* = 0.02; Figure 2A) and a non-significant correlation in the GA + AA genotype group (r = 0.192, *p* = 0.21; Figure 2A). In addition, there was a non-significant correlation between total MUFA and sRAGE with the GG genotype group (r = 0.074, *p* = 0.18), and GA + AA genotype group (r = 0.067, *p* = 0.39), respectively (Supplementary Figure S1B).

When C18:1 was stratified as low C18:1 (<14.51) and high C18:1 (≥14.51), a significant difference in sRAGE was observed for the following RAGE G28S polymorphism: low C18:1/GA + AA versus high C18:1/GG (*p* = 0.015) and high C18:1/GA + AA versus high C18:1/GG (*p* = 0.02) (Figure 2B). Despite the significant effect of stratified C18:1 and sRAGE genotype on plasma sRAGE levels, the interaction effect of these two factors was not significant (% variance explained = 0.50; *p* = 0.32; Figure 2B).

## 4. Discussion

RBC fatty acid composition has been used in several previous studies to reflect dietary fatty acid intake [25]. In a recent study, we reported that CML was negatively associated with RBC saturated fatty acids (SFA) whereas a positive association was observed with polyunsaturated fatty acids (PUFA), suggesting that the concentration of plasma CML may be affected by the relative abundance of fatty acids in RBC membranes [20]. In this study, we investigated the association between different classes of RBC fatty acids and sRAGE in a healthy cohort. We hypothesised that MUFA promotes sRAGE levels which could be influenced by G28S polymorphism in healthy adults because MUFA-rich diets have been associated with a reduction in inflammation and oxidative stress.

In humans, altered circulating sRAGE concentrations have been linked to disease status or risk factors. For example, previous studies showed lower sRAGE levels in subjects with metabolic syndrome [26], obesity [27], and prediabetes [28]. In contrast, elevated levels of sRAGE have been associated with chronic diabetes [29,30] and people with diminished kidney functions [31]. These conflicting data suggest that the potential prognostic value of sRAGE as a marker of disease and the occurrence of adverse events seem to be evident in chronic disease conditions but not in healthy populations. Therefore, in healthy individuals, the increased levels of circulatory sRAGE would be presented as a decoy that would bind AGEs and prevent them from activating RAGE in cells [6,7]. High levels of circulating AGEs have been associated with oxidative stress and chronic inflammation hence increased risk of diabetes and heart disease in humans [2,3,5,6]. To counteract these accumulated AGE levels, the body’s defense system adapts by using different mechanisms such as the glyoxalase system (Glox I and II), AGE receptor-mediated (AGER1 and AGER2) degradation of AGE, and elevation of sRAGE to restrict AGE toxicity thereby reducing oxidative stress and inflammation [4,32]. As sRAGE does not transduce signal upon ligand binding it acts as a decoy molecule to restrain the RAGE/ligand-induced cell activation that is associated with unhealthy metabolic conditions. However, sRAGE level decreases with aging, resulting in suppression of the antioxidant defense system and an increase in the pro-oxidant mechanism which further increases AGE level and reactive oxygen species leading to tissue injury [33,34].

We previously reported that RBC MUFA did not show any significant association with CML, glyoxal, or fluorescent AGE levels [20]. Similarly, in the same cohort, RBC C18:1 showed no statistically significant association with CML (r = 0.020, *p* = 0.397) and glyoxal (r= −0.076, *p* = 0.161) and only a weak non-significant negative correlation for fluorescent AGEs (r= −0.112, *p* = 0.072), respectively. In this study, total MUFA did not show any association with sRAGE levels, however a positive correlation between C18:1 and sRAGE was observed which remained significant after multiple regression analysis adjusted for age, BMI, and gender. In this study, C18:1 constituted approximately 68% of the total MUFA.

In a randomised controlled study, subjects with metabolic syndrome on 12-week high MUFA diets reduced serum AGEs and RAGE mRNA expressions but increased AGER1 and Glox 1 mRNA expression compared to a high SFA diet [15]. In a 4-week cross-over design study, the Mediterranean diet, rich in MUFA, modulated redox-state parameters by reducing AGE levels and increased AGER1 and Glox 1 mRNA expression when compared with the SFA diet in elderly humans [35]. In the CORDIOPREV study, patients with previous cardiovascular events and with type 2 diabetes who were on the Mediterranean diet for 5 years showed a reduction in serum AGEs and an increase in the expression of the AGER1 gene [36]. Our study did not show any association between MUFA and CML/AGEs which is in contrast with previous studies [15,35,36] that could be attributed to the dietary AGE intake. In previous studies, high MUFA diets contained lower AGE levels whereas in our study the participants were on habitual diets, and AGE levels in diets were not assessed.

Previous studies have shown that the G82S polymorphism enhances ligand binding affinity and is associated with increased NFκB activation and inflammatory gene expression [7,8,37]. In addition, the G82S polymorphism is associated with reduced levels of sRAGE, where GA or AA genotypes are reported with low plasma levels of sRAGE compared to the dominant homozygous (GG) genotype [7]. However, how the ligand binding and sRAGE levels are altered by G82S polymorphism is unknown. In our study, the combined AG and AA genotype showed significantly (*p* = 0.001) low levels of plasma sRAGE when compared with GG genotype. Furthermore, in this study, C18:1 showed a non-significant correlation with GA + AA genotype and a positive correlation with the GG genotype. When the C18:1 level was stratified, sRAGE remained unchanged in the GA + AA genotype however, the GG genotype showed an increasing trend in sRAGE levels. In the high C18:1 (≥14.51) group, the plasma sRAGE was significantly higher (*p* = 0.02) in the GG genotype when compared with the GA + AA genotype. The lack of any significance in the GA +AA could be partially attributed to the smaller number of subjects in this group.

**Figure 3:**
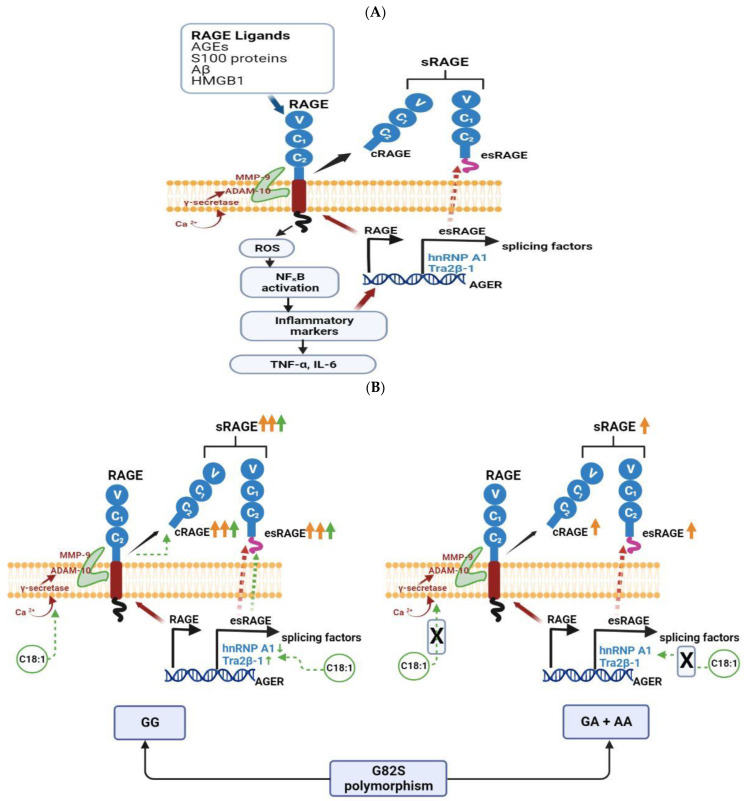
(**A**) Schematic mechanisms of total sRAGE generation. Full-length membrane-bound RAGE consists of an intracellular tail, a transmembrane helix, and an extracellular domain formed by the immunoglobulin-like subdomains V, C1, and C2. When a ligand binds to the extracellular domain, it initiates intracellular signalling that leads to the generation of ROS and to the activation of the transcription factor NF-κB which induces gene expression of pro-inflammatory cytokines such as TNF-α, IL-6, and of RAGE itself. The upregulation of MMP-9 and ADAM-10, which cleave membrane-bound RAGE, liberates the extracellular portion (cRAGE). Additionally, intracellular calcium mobilisation regulates γ-secretase activity that upregulates ADAM-10 which further releases cRAGE. Alternative splicing of the RAGE gene (*AGER*) leads to the generation of esRAGE and contributes to the sRAGE pool. Two splicing factors, heterogenous nuclear ribonucleoprotein A1 (hnRNP A1) and Transformer 2β-1 Tra2β-1), regulates the ratio between the full-length membrane-bound RAGE and esRAGE by exerting antagonistic effects on the selection of alternative exons. (**B**). Proposed mechanism by which oleic acid (C18:1) affects plasma sRAGE levels and its interaction with G82S polymorphism. G82S polymorphism causes a glycine (G) to serine (A) substitution at amino acid number 82 of the ligand-binding V-domain in the extracellular domain that enhances ligand binding to AGEs among others that are associated with increased NF-κB activation and inflammatory gene expression. In turn, this RAGE variant (GA and AA genotypes) cleaves lower levels of sRAGE compared to the wild type (GG genotype). It is plausible that in the GG genotype, the presence of C18:1 in the transmembrane promotes (i) MMP-9 and (ii) ADAM-10 via induced γ-secretase through intracellular calcium mobilisation which, in turn, regulates the intermembrane proteolysis of RAGE and generates cRAGE hence increased total sRAGE levels. Another plausibility may be that C18:1 promotes esRAGE resulting from alternative splicing of RAGE pre-mRNA by upregulating Tra2β-1 and downregulating hnRNP A1 genes.

To our knowledge, this is the first study that shows a relationship between C18:1 and the G82S polymorphism with regard to sRAGE level in plasma. The results suggest that the G82S polymorphism when homozygous for G codes for a form of RAGE that has a greater propensity to become sRAGE by cleavage from the cellular membrane and that this process may be further influenced by increased C18:1 level. The exact mechanism as to how sRAGE levels are altered by G82S polymorphism is unknown. However, it could be plausible that C18:1 may either stimulate the proteolytic enzymes responsible for cRAGE production or favour esRAGE from alternative splicing of AGER genes hence increasing total sRAGE levels in the GG genotype which does not occur in the GA + AA genotype. At the cell surface, cRAGE is produced by proteolytic cleavage of RAGE which occurs at the boundary between its extracellular and transmembrane [4]. Two metalloproteases, (MMP-9 and ADAM-10), G protein-coupled receptors, intracellular calcium mobilisation, and elevation of cAMP levels have been associated with the generation of the cleaved form of RAGE [4,38,39] (Figure 3A). In addition, cRAGE is also produced through the regulation of intramembrane proteolysis of RAGE which is catalysed by ADAM-10 and γ-secretase, in which calcium is a fundamental regulator of sRAGE processing [40] (Figure 3A). In a previous study, the presence of C18:1 promoted MMP-9 secretion through a PKC, Src, and EGFR-dependent pathway suggesting that C18:1 may have a crucial role in the invasion process and metastasis in breast cancer [41]. In addition, C18:1 has previously been shown to induce intracellular calcium mobilisation [42,43] and regulate γ-secretase activity [44,45]. Taken together, in our study, it is plausible that in the GG genotype, C18:1 promotes (i) MMP-9 and (ii) ADAM-10 via induced γ-secretase through intracellular calcium mobilisation which, in turn, regulates the intermembrane proteolysis of RAGE and generates cRAGE hence causing increased total plasma sRAGE levels (Figure 3B).

The role of C18:1 on preferring esRAGE resulting from alternative splicing of RAGE needs to be elucidated. The alternative splicing of RAGE pre-mRNA generates more than 20 splice variants, the two most abundant variants include full-length membrane-bound RAGE and esRAGE (Figure 3A). Two splicing factors, namely heterogenous nuclear ribonucleoprotein A1 (hnRNP A1) and Transformer 2β-1 Tra2β-1), regulates the ratio between the full-length membrane-bound RAGE and esRAGE by exerting antagonistic effects on the selection of alternative exons [4,46]. In addition, previous studies have shown that impaired glucose metabolism may affect the regulation of esRAGE levels by upregulating the hnRNP A1 and down-regulation of Tra2β-1, resulting in a decrease in esRAGE expression [46]. Several studies have supported the role of the Mediterranean diet and C18:1 in glucose metabolism [47,48,49] hence it can be speculated that C18:1 could in part favour esRAGE expression by upregulating Tra2β-1 and downregulating hnRNP A1 (Figure 3B). However, esRAGE accounts for less than 25% of total circulating sRAGE therefore any contribution of esRAGE to the increased total plasma sRAGE will be relatively small [50].

The strength of our cross-sectional study is that it provides novel information on the association between individual RBC fatty acids on AGE metabolism by measuring the association with sRAGE. The study focused on individuals who were on normal diets rather than diets supplemented with higher specific fatty acid levels, to reflect the effects of habitual diets in healthy Australians. However, it would be useful to further explore the role of specific fatty acids with respect to specific AGEs, which could provide direct insights into the role of fatty acids in AGE distribution, accumulation, and metabolism. We observed a positive correlation of C18:1 with sRAGE and further a positive correlation with the GG genotype suggesting that C18:1 could be a potential therapeutic approach in AGE metabolism. In this study, total sRAGE was measured however, it will be useful to separately measure cRAGE and esRAGE to gain a better understanding of the exact pathway responsible for increased total sRAGE.

## 5. Conclusions

In conclusion, our study showed a non-significant correlation between total MUFA with sRAGE except for C18:1 which showed a positive correlation that remained significant after a stepwise multivariate regression analysis adjusted for age, BMI, and gender. In a univariate analysis, a positive significant correlation between C18:1 and sRAGE in GG genotype was observed but not in the GA + AA genotype. When C18:1 was stratified as low C18:1 (<14.51) and high C18:1 (≥14.51), a significant difference was observed for low C18:1/GA + AA compared to high C18:1/GG and high C18:1/GA + AA compared to high C18:1/GG. In this study, healthy adults were on a habitual diet and not on any dietary supplementation thus the RBC C18:1 and other FA levels were from their normal diet which could partially contribute to the weak association however these relationships were statistically significant. Our study suggests that increased levels of C18:1 could be a potential therapeutic approach in increasing sRAGE in those with GG genotype and play a role in modulating AGE metabolism thus reducing oxidative stress/inflammation in this sub-group. The possibility that increased oleic acid intake through dietary supplementation may increase sRAGE to a greater extent in those homozygous for the G allele G82S polymorphism of sRAGE relative to A carriers needs to be verified in a larger cross-sectional study and well-designed controlled interventions.

## Figures and Tables

**Table 1:** Clinical and laboratory characteristics of the study cohort (n = 172).

Variables	Value
Male sex, n (%)	36 (20.7)
Age (years)	53.74 ± 0.61
BMI (kg/m^2^)	26.52 ± 0.37
***Lipid profile*** *s*	
Total cholesterol (mmol/L)	5.42 ± 0.07
HDL cholesterol (mmol/L)	1.65 ± 0.03
LDL cholesterol (mmol/L)	3.27 ± 0.06
Triglycerides (mmol/L)	1.10 ± 0.04
***Glycation biomarkers***	
Glucose (mmol/L)	4.54 ± 0.07
Glyoxal (nmol/L)	13.93 ± 0.24
Fluorescent AGEs (AU/mL)	2620.43 ± 63.85
*N*^ε^-Carboxymethyllysine (μmol CML/mol lysine)	48.94 ± 0.51
sRAGE (pg/mL)	659.12 ± 22.49

All values are means (±SE) except for quantitative variables (expressed as n and %).

**Table 2:** Glycation biomarkers based on RAGE G82S polymorphism.

	RAGE G82S Polymorphism		*p*-Value
	AG + AA (n = 20)	GG (n = 152)	
Glucose (mmol/L)	4.76 ± 0.16	4.51 ± 0.07	0.155
Glyoxal (nmol/L)	15.34 ± 0.75	13.75 ± 0.25	0.057
Fluorescent AGEs (AU/mL)	2861.58 ± 242.80	2588.70 ± 64.50	0.289
N*^ε^*-Carboxymethyllysine (μmol CML/mol lysine)	50.81 ± 1.58	48.77 ± 0.53	0.271
sRAGE (pg/mL)	517.38 ± 35.77	677.77 ± 24.57	0.001

Values are presented as mean ± SEM.

## Data Availability

Data will be uploaded to a publicly available repository upon acceptance of the manuscript.

## Supplementary Materials

The following supporting information can be downloaded at: https://www.mdpi.com/article/10.3390/cells12121662/s1, Table S1: Red blood cell fatty acid composition in the study cohort. Figure S1: (A) Regression analysis of monounsaturated fatty acid (MUFA) and oleic acid (expressed as % of total RBC fatty acids) with sRAGE. (B) Regression analysis of monounsaturated fatty acid (MUFA) and oleic acid (expressed as % of total RBC fatty acids) with sRAGE stratified by RAGE.genotype (AG + AA; GG).
