# Supplementary material for: Oleic Acid Status Positively Correlates with the Soluble Receptor for Advanced Glycation End-Products (sRAGE) in Healthy Adults Who Are Homozygous for G Allele of RAGE G82S Polymorphism
Source: Cells. 2023 Jun 19;12(12):1662. doi: 10.3390/cells12121662 (PMC10297244; doi:10.3390/cells12121662)
Supplement: Supplementary file 1 [file cells-12-01662-s001.zip › cells-2294509-supplementary-/Supplementary Table 1-Final.pdf]

Supplementary Table S1. Red blood cell fatty acid composition in the study cohort

RBCs Fatty acid	Mean ± S.E* (interquartile range)
**Saturated fatty acids**	
Myristic acid (C14:0)	0.31 ± 0.00 (0.14 – 0.57)
Pentadecyclic acid (C15:0)	0.40 ± 0.01 (0.13 – 0.75)
Palmitic acid (C16:0)	21.18 ± 0.20 (17.60 – 30.69)
Stearic acid (C18:0)	17.67 ± 0.14 (15.32 – 25.47)
Arachidic acid (C20:0)	0.67 ± 0.01 (0.47 – 1.08)
Behenic acid (C22:0)	2.44 ± 0.02 (1.71 – 3.69)
Tricosylic acid (C23:0)	0.37 ± 0.01 (0.24 – 0.68)
Lignoceric acid (C24:0)	8.53 ± 0.07 (6.52 – 11.90)
Total SFA	51.57 ± 0.46
**Monounsaturated fatty acids**	
Palmitoleic acid (C16:1n-9)	0.29 ± 0.01 (0.13 – 1.54)
Oleic acid (C18:1n-9)	14.49 ± 0.08 (12.03 – 17.90)
Nervonic acid (C24:1n-9)	6.43 ± 0.06 (4.60 – 8.74)
Total MUFA	21.21 ± 0.15
**Polyunsaturated fatty acids**	
Linoleic acid (C18:2n-6)	7.15 ± 0.10 (2.40 – 9.58)
γ-Linolenic acid (C18:3n-6)	0.08 ± 0.00 (0.00 – 0.12)
α-Linolenic acid (C18:3n-3)	0.08 ± 0.00 (0.00 – 0.22)
Dihomo-γ-linolenic acid (C20:3n-6)	1.25 ± 0.03 (0.12 – 2.02)
Arachidonic acid (C20:4n-6)	10.82 ± 0.23 (1.21 – 15.53)
Eicosapentanoic acid (C20:5n-3)	0.93 ± 0.06 (0.04 – 4.57)
Docosadienoic acid (C22:2n-9)	0.18 ± 0.01 (0.10 -0.49)
Docosapentaenoic acid (C22:5n-3)	2.08 ± 0.06 (0.19 – 3.97)
Docosahexaenoic acid (C22:6n-3)	4.64 ± 0.13 (0.66 – 8.50)
Total PUFA	27.21 ± 0.62

* expressed as % of total RBC fatty acids.
