# Supplementary figures and images for: Oleic Acid Status Positively Correlates with the Soluble Receptor for Advanced Glycation End-Products (sRAGE) in Healthy Adults Who Are Homozygous for G Allele of RAGE G82S Polymorphism
Source: Cells. 2023 Jun 19;12(12):1662. doi: 10.3390/cells12121662 (PMC10297244; doi:10.3390/cells12121662)

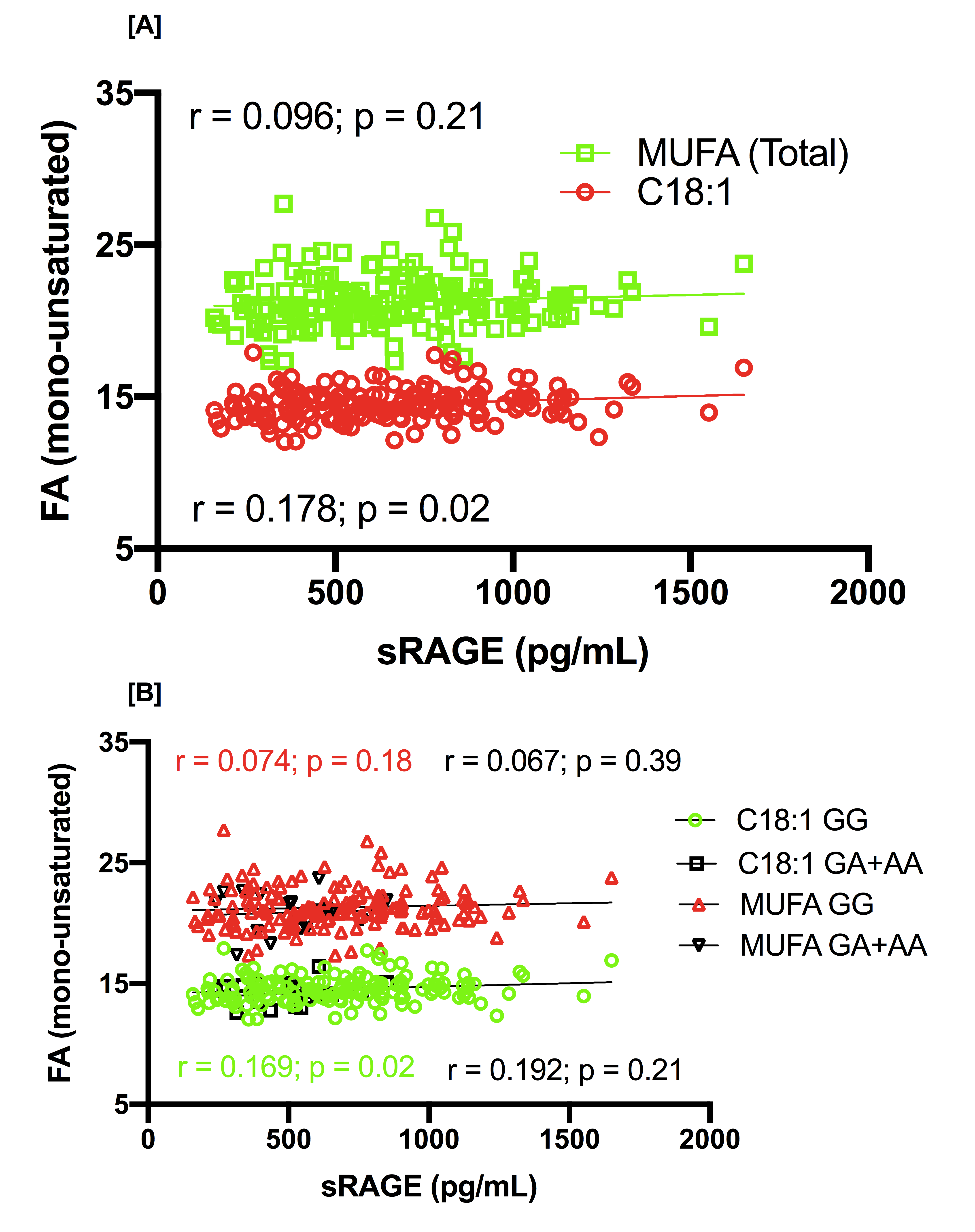

Supplement: Supplementary file 1 [file cells-12-01662-s001.zip › cells-2294509-supplementary-/Supplementary Figure.tiff]
